# How does engagement in society in adolescence affect educational attainment and employment in early adulthood: A prospective cohort study

**DOI:** 10.1371/journal.pone.0249312

**Published:** 2021-04-01

**Authors:** Vivi Just-Noerregaard, Johan Hviid Andersen, Ellen Aagaard Nohr, Jesper Medom Vestergaard, Trine Nohr Winding

**Affiliations:** 1 Department of Occupational Medicine, Danish Ramazzini Centre, University Research Clinic, Regional Hospital West Jutland, Herning, Denmark; 2 Research Unit for Gynaecology and Obstetrics, Institute for Clinical Research, University of Southern Denmark, Odense, Denmark; 3 Centre of Women’s, Family and Child Health, University of South-Eastern Norway, Kongsberg, Norway; Queens University Belfast, IRELAND

## Abstract

**Introduction:**

Educational attainment and employment are essential for young people to develop the skills needed to participate in society and maintain a stable connection to the labour market in adult life. The objective of this study was to examine associations between engagement in society, measured by leisure time activities and part-time work in mid and late adolescence and educational attainment and employment in early adulthood.

**Method:**

A cohort of Danish young people born in 1989 was followed in a prospective study with questionnaires in 2004 (n = 3,054) and 2007 (n = 2,400) where information on leisure time activities and part-time work was collected. Information on connection to education and work was collected from a register of social benefits when participants were 25–29 years old and divided into high and low connection. The associations were examined using logistic regression and stratified by gender and childhood socioeconomic groups.

**Results:**

Part-time work was, both in mid (OR: 1.7 [95% CI 1.3; 2.2]) and late (1.9 [1.4;2.6]) adolescence, positively associated with connection to education and work. Leisure time activities in mid adolescence were associated with connection to education and work (OR:1.6 [1.2;2.1]). Among men engagement in society showed strongest associations with later connection to education or work in mid adolescence (ORs up to 2.2), whereas the associations for women seemed strongest in late adolescence (ORs up to 2.8).

**Conclusion:**

The study showed that adolescent engagement in society had positive associations with later educational attainment and employment, with stronger impact of part-time work compared to leisure time activities. The study identified differences between genders and the timing of engagement. Associations were consistent across socioeconomic groups.

## Introduction

In Denmark, young people under the age of 30 are challenged to get a job and acquire a stable attachment to the labour market. During the last 10 years, there has been an almost 20% increase in the number of young adults, who neither participate in education, employment nor training (NEET) [1]. In addition, there has been a 30% increase among 15-29-year-olds living on social benefits, compared to before the financial crisis in 2008 [2]. The negative consequences of youth unemployment persist into adulthood [3]. Having a stable job has shown to be advantageous in relation to remain connected to society throughout adulthood [4]. In 2018, Denmark spent 28% of the Gross Domestic Product on societal expenditures [5]. Due to a growing and aging population, there is an increasing need to maximise labour market participation, and several labour areas are currently unable to get sufficient employees, thus losing revenue. If young adults do not have the adequate skills to take part in the labour market, welfare societies such as the Danish are challenged with both a deprivation of labour and with increased expenditures in terms of social and unemployment benefits [4, 6].

Young adults who are not included in society at the same level as their peers are described with various terms as disconnected [7], marginalized [8, 9], and socially excluded [10]. Yet, these terms describe a complex and often multi-dimensional state where young adults, because of their life trajectories, lack access to certain resources, commodities, and services. This affects both the quality of life of the young adults and the equity and cohesion of society as a whole [7, 8, 10]. Lost connection is a continuum of both duration and severity. Some young adults lose their connection for long periods of time and may never regain connection, whereas others recommence societal connection after a shorter period. For some young adults, a period of disconnection can have life-altering consequences [11].

NEETs between the ages of 16–24 have been shown to have a lower degree of employment 8 years later (47%), compared to adolescents attaining education or employment in the same period (76%) [3]. The more disconnected a person is in early adulthood, the more likely they are to receive social benefits or disability pensions 8 years later. Being disconnected increases the risk of poor somatic or mental health, delinquency, and premature death [9, 12, 13].

The risk of disconnection is negatively influenced by poor family background, persisting throughout life with lower education, employment, and income as a result [7, 14]. Education increases the ability to achieve high-paying jobs, improve living conditions, and the quality of health-related choices [15]. Half of the disconnected 18-24-year-olds in Denmark had disconnected parents [9]. Disconnection varies between genders with more men being disconnected than women and often to a greater extent [9, 16]. In order to reduce the personal and societal costs of unemployment, and improve the future demands for labour, it is imperative to investigate factors and mechanisms associated with disconnection.

The degree of young adults’ risk of disconnection is influenced by their human capital. Human capital is the skills, traits, and abilities of a person, which shapes his or her position in society [17]. It comprises of cultural, social, and economic capital, all of which are important and relevant in personal development [18]. Economic capital mostly reflects the ability to perform work, which increases one’s economic value. Social and cultural capital refers to the relationships and influences individuals use to engage in society. Formal education is not the only way to invest in human capital. Learning and training outside the school, especially in a work setting or extracurricular activities also improve human capital [19].

### Adolescent engagement in society

Part-time work (PTW) and leisure time activities (LTA) are examples of activities where adolescents are engaged in society.

PTW is paid employment whilst the employee is attaining education. Around 18% of Danish 13-year-olds have a PTW, rising to 70% by the age of 17 [19]. Adolescent PTW has been found to shape life skills, work ethics, self-efficacy, career planning, and aspirations [19–22]. Stressors experienced in PTW have been found to buffer the effect of more severe stressors later in life [23]. Danish adolescents who worked 20–30 hours per month had less school absence and better grades in Danish, Math, and English when finishing 9^th^ grade [19]. Contrary, some studies found PTW was associated with lower levels of educational completion, lower grades, risk of dropping out or reduce enrolment into tertiary education [24–26].

LTA are chosen for enjoyment, relaxation, or other emotional reasons. The specific type of LTA and its societal context affect social, educational, physical, and civic development and shape young people’s future lives [27]. Organized and adult supervised LTA tends to increase later life outcomes positively e.g. satisfaction with life and physical and mental health [28]. Participation in activities such as scouts and church clubs has been associated with positive adult outcomes regarding economic status, family situation, health and well-being, and citizenship [28, 29]. In contrast, adolescent LTA performed alone or in unstructured settings, is associated with the risk of disconnection, crime, or unemployment in early adulthood [27, 29].

A socioeconomic gradient is found in adolescents’ participation in LTA. Adolescents from higher socioeconomic position are found to be more engaged in LTA [30, 31]. This can be explained in part by stronger stimulation from parents with higher socioeconomic position, they are more likely to have active lifestyles themselves and influence their children through role modelling or prioritizing the development of their children’s social resources through LTA [30]. Contrary, an American study found that adolescents with less educated parents and from lower household income families worked more hours than their more advantaged counterparts during high school [32].

Adolescents ability to engage in PTW and many types of LTA are negatively associated with pre-existing disabilities due to physical or psychological constraints and potentially reduced accessibility [33]. Mental disorders, for example, depressive symptoms can also be associated with the adolescent’s ability to engage in out-of-school activities regardless of it being work or leisure activities. Blanco and Barnett have described these adverse outcomes among college students with depressive symptoms [34]. Disabilities and depressive symptoms in adolescence are also both associated with the ability to achieve employment and educational attainment in later life [35–37]. A Danish study show that 46% of young adults with a handicap have not completed a secondary education by the age of 25 [38]. In comparison, for adolescents without handicaps it is 15% who had not completed secondary education by the age of 25 [38]. Employment rates are also lower for people with handicaps [39].

In order to reduce the number of disconnected young adults, it is important to identify tangible actions that can improve the connection to society. When adolescents participate in PTW and LTA, they inevitably engage in the wider society. In return, they develop new skills and traits, and these competencies may buffer the risk of disconnection. However, research on adolescent engagement in society measured by PTW and LTA and connection to education and employment in early adulthood is scarce.

The primary aim of this study was to examine whether skills and traits attained through engagement in society (PTW and LTA) in mid and late adolescence were associated with connection to education or work in early adulthood. In addition, the study aimed to investigate to what degree the possible associations varied across gender and socioeconomic groups.

## Materials and methods

### Design and population

**Fig 1:**
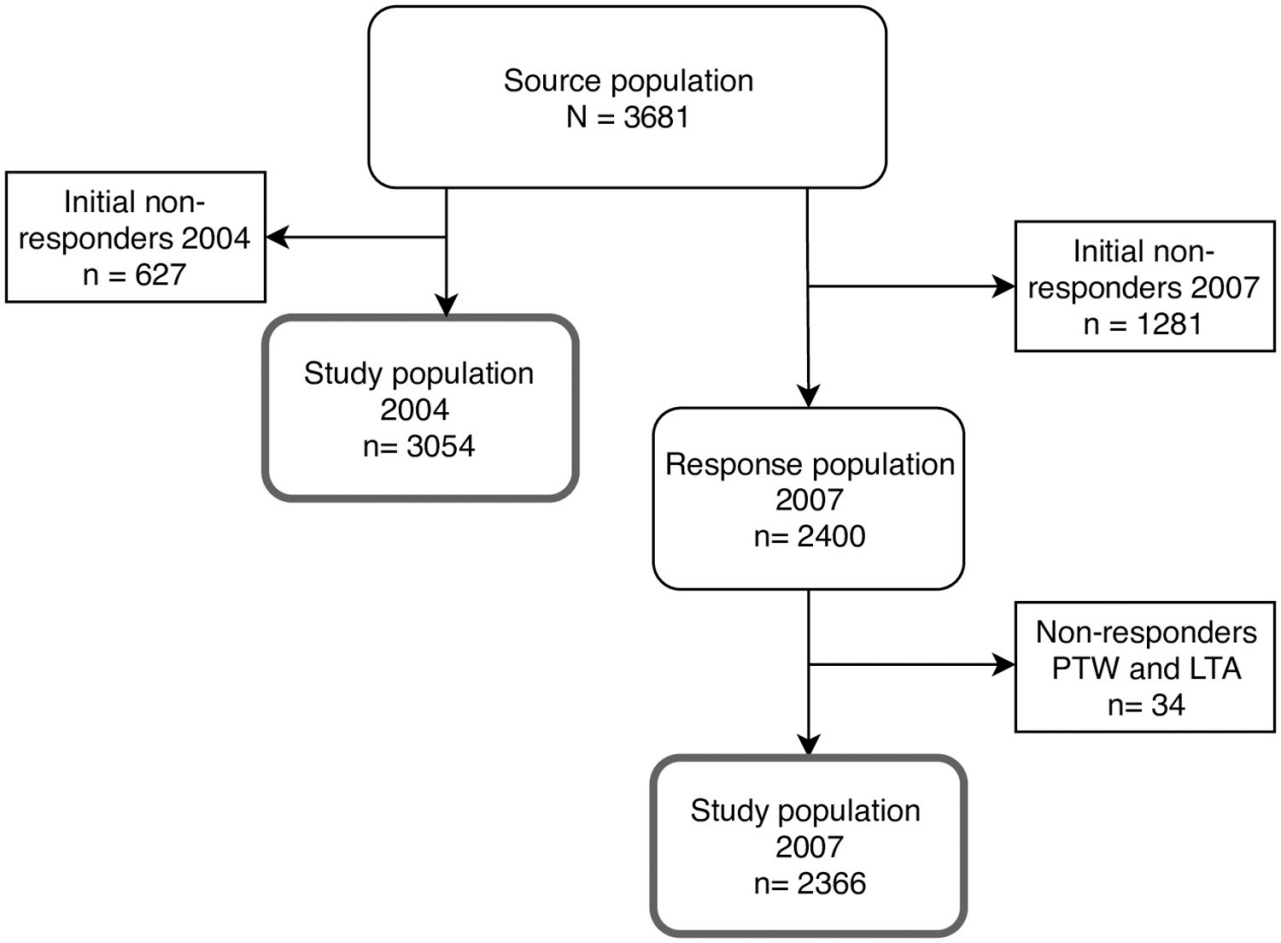
Inclusion into study populations for mid and late adolescence. Participants needed to respond to either part-time work or leisure activities to be included in a study population.

The study was a prospective follow-up study. Data was collected as part of the VestLiv Cohort, an ongoing longitudinal Danish cohort study following a complete regional cohort of young people born in 1989 who lived in the western part of Denmark when the cohort was established in 2004 exploring social inequality in relation to health and morbidity [40]. The source population comprised of 3,681 young people. All potential responders in the 2004 survey have consecutively been invited to participate in later waves of the questionnaires. In the first wave of questionnaires in 2004, 3,054 (83%) participated. In the second wave, 65% (n = 2,400) of the source population participated. Besides information from the participants, information was obtained from the parents via a questionnaire in the first wave. A more thorough description of recruitment and data collection in VestLiv has been presented elsewhere [41]. Participants who answered questions about PTW and/or LTA in the waves of 2004 and/or 2007 were included in this study. They formed two separate study populations representing mid and late adolescence. The number of responders varied in the two waves: 2004 (n = 3,054) and 2007 (n = 2,400). Responses to at least one question about PTW or LTA were obtained from all 3,054 participants in the first wave and 2,366 participants in the second wave. Fig 1 illustrates the inclusion in both study populations.

Furthermore, register data was obtained from Statistics Denmark. Using the personal identification number (CPR number), which is given to every citizen in Denmark, a linkage between the participant and the parents was created through a unique family ID number, and subsequently between the various registers and databases using the CPR numbers [42].

### Definition of outcome

The outcome "Connection" was defined, as the amount of connection to education and employment from the age of 25 to the age of 29. Lack of connection was measured using information from the Danish Register for Evaluation of Marginalization (DREAM), a longitudinal database that contains weekly registrations of the extent and kind of social benefits such as unemployment benefits, sickness benefits, and disability benefits that Danish citizens may receive [43]. All types of social benefits were categorized as lack of connection to education and employment, except maternity leave, educational grants, and supplementary unemployment benefits, which is available to job seeking individuals who are employed less than 30 hours per week. Participants who had not received any benefits were also registered in DREAM and were categorized as receiving no benefits. The participants were followed in DREAM from 1^st^ of January 2015 to 2^nd^ of December 2018 (approximately 4 years) and were dichotomized into low or high connection to education or employment based on the total number of weeks receiving social benefits [44] over the 4 year period. Participants with ≤ 52 weeks of receiving social benefits were coded as having "high connection", those with >52 weeks of receiving social benefits were coded as having "low connection". DREAM data was available for the last 4 weeks of December 2018 at the time of analysis. This meant that participants born in December 1989 (n = 234) potentially could lack up to 4 weeks of registered disconnection.

### Definition of exposures

The exposure variables contained in the term " engagement in society" were based on questionnaire information from 2004 and 2007 about PTW and LTA. Engagement in society was both examined in mid adolescence (15 years) and late adolescence (18 years) to identify if the timing of engagement played a role for future connection to education and employment.

LTA were activities like after school clubs, scout troops, sports clubs, bands, or voluntary activities such as church groups or theatres. Participants were categorized as having “high” LTA if they had marked daily or weekly LTA activities in at least two of the four categories of activities: 1) after-school classes/scout/youth club, 2) choir/orchestra/music group, 3) sports club or 4) other clubs. Otherwise, participants were categorized as having “low” LTA.

PTW was dichotomized into “engaged” or “not engaged” if they had a job regardless of the number of work hours per week. Additionally, PTW was recoded into a categorical variable based on the number of work hours per week “0 hours/w” “1–12 hours/w” “>12 hours/w”. The cut-off values were based on studies indicating that higher amounts of work hours whilst still at school may have a negative effect on educational attainment [21, 26] and The Danish Working Environment Authority, which allows adolescents in compulsory school to work 2 hours per school day and up to 12 hours per week [45, 46].

### Other variables

#### Equivalised childhood household income

Equivalised childhood household income was generated by averaging the mean of the yearly household income from the participants were 7–10 years old if data was available for at least 3 of the 4 years. It was generated using data from the national family income register [47]. Equivalised household income is a weighted measure taking household size and composition into account. The weighing was based upon the OECD-modified Scale where the first adult counts 1.0, other adults and children over 14 years count 0.5, and children (under 15 years) count 0.3 [48]. Yearly household income was divided by the weighing factor each year. We categorized the variable into low, medium, and high equivalised household income, grouped by the 33.3^rd^ and 66.7^th^ percentiles within the source population.

#### Parents educational level

The highest level of completed education of the parents was assessed in 2004 to reflect the educational status of the participants’ childhood home. The variable was generated with data from the Danish National Registry of highest attained education. The primary data sources to the registry were the Student Register and the Qualification Register [49]. It was categorized into 3 levels based on completed education: high (>13 years of education), medium (10–13 years), and low (<10 years) as recommended by Jensen and Rasmussen [50].

#### Subjective social status

Subjective social status was measured in 2004 by questionnaire using The MacArthur Scale of Subjective Social Status [51, 52], which is a ladder of ten steps, where participants mark their family compared to other families in Denmark. A higher mark indicated a higher subjective social status. The scores were categorized at the 33.3^rd^ and 66.7^th^ percentile into three levels: high, medium, and low. The measure has been identified to be relatively stable during adolescence [53].

#### Health conditions

In 2004, the parents answered, as legal guardians of the participants, whether the participants had any chronic conditions. Subsequently, the participants answered the same question themselves in 2007. All chronic conditions were evaluated by the main author and dichotomized into, “no chronic diseases” and “yes”. Chronic diseases included all conditions expected to impact the ability of societal engagement or connection to education and employment. Examples of chronic diseases are attention deficit/hyperactivity disorders (ADHD), autism, asthma, or cardiac diagnoses. Contrary to e.g. eczema, hay fever, backaches, or hypermobile joints which are not expected to affect the outcome or exposure of the study.

#### Depressive symptoms

Depressive symptoms were assessed with an abbreviated 4 item version of the Center for Epidemiological Studies Depression Scale for Children (CES-DC) [54]. The scale measures the level of current depressive symptoms. All questions were answered on a four-point Likert scale with the options: “not at all”, “a little”, “some” and “quite a lot”. Each answer was scored between 0 and 3, equal to a maximum score of 12, with high values corresponding to having depressive symptoms. In this study, depressive symptoms were dichotomized into absent or present at a cut point of ≥4 because the study examined a young and healthy population who were anticipated to have good mental health.

#### Self-rated health

Self-rated health is a valid measure of physical health status, and a strong predictor for future outcomes such as morbidity and mortality [55, 56]. Self-rated health was assessed by a single question on a five-point Likert scale from the 36-Item Short-Form Health Survey (SF-36) [57]. Self-rated health was dichotomized into; "good" self-rated health if participants rated their health as excellent or very good or "poor" self-rated health if participants rated their health as good, less good, or bad.

### Statistical methods

Characteristics of the study populations and connection to education and employment were displayed according to gender and differences were tested applying Pearson chi-squared test (Table 1). Univariate analyses were performed to describe associations between each covariate and connection to education and employment in early adulthood (Crude analyses, Tables 2 and 3).

**Table 1:** Characteristics of the two study populations, mid adolescence, n = 3,054 and late adolescence, n = 2,366.

Source population		Women (%)	Men (%)					
N = 3681		1777 (48)	1904 (52)					
	Mid adolescence (15 years)				Late adolescence (18 years)			
		Women	Men	Total		Women	Men	Total
**Study population**	**n = 3054**	**1536 (50)**	**1518 (50)**		**n = 2366**	**1277 (54)**	**1089 (46)**	
Leisure time activities	n = 2999				n = 2359			
High (%)		525 (35)	503 (34)	1028 (34)		157(12)	140 (13)	297 (13)
Low (%)		980 (65)	991 (66)	1971 (66)		1118 (88)	944 (87)	2062 (87)
Part time work	n = 3042				n = 2332			
Yes (%)		1004 (66)	1011(67)	2015 (66)		829 (66)	484 (45)	1313 (56)
No (%)		527 (34)	500 (33)	1027 (34)		437 (34)	582 (55)	1019 (44)
Part time work (work hours/week)	n = 2963				n = 2332			
> 12 hours (%)		95 (5)	169 (9)	264 (7)		172 (14)	90 (8)	262 (11)
1–12 hours (%)		868 (49)	804 (42)	1672 (45)		657 (52)	394 (37)	1051 (45)
0 hours (%)		527 (30)	500 (29)	1027 (28)		437 (35)	582 (55)	1019 (44)
Equivalised childhood household income per family member	n = 3000				n = 2331			
High (> 13.400 EUR) (%)		535 (36)	511 (34)	1046 (35)		449(36)	397(37)	846 (36)
Medium (10.980–13.400 EUR) (%)		510 (34)	512 (34)	1022 (34)		435 (35)	364 (34)	799 (34)
Low (< 10.980 EUR) (%)		452 (30)	480 (32)	932 (31)		373 (30)	313 (29)	686 (29)
Parents highest educational level	n = 2939				n = 2331			
>13 years (%)		559 (38)	609 (42)	1168 (40)		498 (40)	457 (43)	955 (41)
10–13 years (%)		728 (49)	672 (46)	1400 (48)		609 (48)	498 (47)	1107 (48)
< 10 years (%)		190 (13)	181 (12)	371 (13)		152 (12)	117 (11)	269 (12)
Subjective Social Status^	n = 2971				n = 2105			
High (%)		867 (58)	969 (66)	1836 (62)		668 (58)	631 (66)	1299 (62)
Medium (%)		408 (27)	318 (22)	726 (24)		314 (28)	208 (22)	522 (25)
Low (%)		225 (15)	184 (13)	409 (14)		162 (14)	122 (13)	284 (14)
Chronic disease	n = 2580				n = 2352			
No (%)		1166 (90)	1151 (90)	2317 (90)		1067 (84)	948 (88)	2015 (86)
Yes (%)		131 (10)	132 (10)	263 (10)		203 (16)	134 (12)	337 (14)
Depressive symptoms*	n = 3000				n = 2348			
Absent (%)		1116 (74)	1221 (82)	2337 (78)		755 (59)	820 (76)	1575 (67)
Present (%)		401 (26)	262 (18)	663 (22)		516 (41)	257 (24)	773 (33)
Self-rated health**	n = 3034				n = 2355			
Good (%)		1057 (69)	1203 (80)	2260 (75)		721 (57)	788 (73)	1509 (64)
Poor (%)		472 (31)	302 (20)	774 (26)		550 (43)	296 (27)	846 (36)
Connection to education and employment 25–29 years	n = 3054				n = 2366			
High (≤52 weeks of disconnect.) (%)		1300 (85)	1351 (89)	2651 (87)		1106 (87)	969 (90)	2075 (90)
Low (> 52 weeks of disconnect.) (%)		236 (15)	167 (11)	403 (13)		171 (13)	120 (11)	291 (11)

Percentages may not add to 100 due to rounding to whole integers.

^) Measured in 2004. A value between 1–10. Higher score indicates higher subjective social status. Categorized into low, medium and high subjective social status

*) The cut-off point for depressive symptoms has been changed from the original score of > 2 to a score of more than 3 before being coded with depressive symptoms.

**) Self rated health is rated as: good health: Excellent and very good, poor health: Good, less good and bad.

**Table 2:** Connection to education or employment in early adulthood according to engagement in society in mid adolescence.

Exposures measured in mid adolescence (15 years)	Connection to education and employment—All				Connection to education and employment—Women				Connection to education and employment—Men			
	Crude analyses		Adjusted Model ^		Crude analyses		Adjusted Model ^		Crude analyses		Adjusted Model ^	
	n	OR [95% CI]	n	OR [95% CI]	n	OR [95% CI]	n	OR [95% CI]	n	OR [95% CI]	n	OR [95% CI]
Leisure time activities ^a^	2999		2383		1505		1205		1494		1178	
High (≥2 LTAs/w)		1.6 [1.2;2.0]		1.6 [1.2;2.1]		1.5 [1.1;2.1]		1.5 [1.0;2.2]		1.6 [1.1;2.3]		1.7 [1.1;2.7]
Low (<2 LTAs/w) (ref)		1		1		1		1		1		1
Part time work	3042		2407		1531		1220		1511		1187	
Yes		1.9 [1.5;2.3]		1.7 [1.3;2.2]		1.8 [1.4;2.4]		1.7 [1.6;2.3]		2.0 [1.4;2.8]		1.7 [1.2;2.6]
No (ref)		1		1		1		1		1		1
Part time work by hours	2963		2352		1490		1168		1473		1166	
>12 hours/w		2.1 [1.3;3.2]		1.9 [1.1;3.3]		1.8 [0.9;3.4]		1.5 [0.7;3.2]		2.1 [1.1;3.8]		2.2 [1.0;4.9]
1–12 hours/w		1.9 [1.6;2.4]		1.7 [1.3;2.2]		1.8 [1.4;2.5]		1.7 [1.2;2.4]		2.1 [1.5;3.0]		1.7 [1.1;2.6]
0 hours/w (ref)		1		1		1		1		1		1
Equivalised childhood household income ^b^	3000				1503				1497			
High (> 13.400 EUR)		1.7 [1.3;2.2]				1.5 [1.0;2.1]				1.9 [1.3; 2.9]		
Medium (10.980–13.400 EUR)		1.1 [0.9;1.5]				1.0 [0.7;1.4]				1.3 [0.9;2.0]		
Low (<10.980 EUR) (ref)		1				1				1		
Parents highest edu. level ^c^	2939				1477				1462			
>13 years		2.3 [1.7;3.2]				2.0 [1.3;3.1]				2.7 [1.7;4.2]		
10–13 years		2.2 [1.6;2.9]				1.8 [1.2;2.7]				3.0 [1.9;4.6]		
<10 years (ref)		1				1				1		
Subjective Social Status	2971				1500				1471			
High		2.3 [1.7;3.1]				1.8 [1.2;2.6]				3.1 [2.0;4.6]		
Medium		1.4 [1.0;1.9]				1.1 [0.7;1.6]				2.1 [1.3;3.3]		
Low (ref)		1				1				1		
Chronic disease	2580				1297				1283			
No		2.5 [1.9;3.4]				2.1 [1.4;3.2]				3.2 [2.0;5.0]		
Yes (ref)		1				1				1		
Depressive symptoms	3000				1517				1483			
Absent		1.7 [1.3;2.1]				1.5 [1.1;2.0]				1.8 [1.2;2.6]		
Present (ref)		1				1				1		
Self-rated Health	3034				1529				1505			
Good		1.7 [1.4;2.2]				1.6 [1.2;2.2]				1.7 [1.2;2.4]		
Poor (ref)		1				1				1		

^) Model: adjusted for equivalated childhood income, parents’ highest educational level, subj. social status, chronic diseases, depressive symptoms and self-rated health.

^a)^ Engagement in 2 different types of LTA daily or weekly per week

^b)^ Childhood equivalised household income categorized at the 33^rd^ and the 67^th^ percentile of the source population

^c)^ Highest completed educational level of the biological parents when each participant was 7–10 years old.

**Table 3:** Connection to education or employment in early adulthood according to engagement in society in late adolescence.

Exposures measured in late adolescence (18 years)	Connection to education and employment—All				Connection to education and employment—Women				Connection to education and employment—Men			
	Crude analyses		Adjusted Model ^		Crude analyses		Adjusted Model ^		Crude analyses		Adjusted Model ^	
	n	OR [95% CI]	n	OR [95% CI]	n	OR [95% CI]	n	OR [95% CI]	n	OR [95% CI]	n	OR [95% CI]
Leisure time activities ^a^	2359		2025		1275		1105		1084		920	
High (≥2 LTAs/w)		1.7 [1.1;2.6]		1.4 [0.9;2.3]		2.2 [1.2;4.2]		1.9 [0.9;3.8]		1.2 [0.7;2.3]		1.0 [0.5;2.0]
Low (<2 LTAs/w) (ref)		1		1		1		1		1		1
Part time work	2332		2001		1264		1095		1065		906	
Yes		2.2 [1.7;2.8]		1.9 [1.4;2.6]		2.7 [1.9;3.8]		2.3 [1.6;3.4]		1.9 [1.2;2.8]		1.7 [1.1;2.7]
No (ref)		1		1		1		1		1		1
Part time work by hours	2332		2004		1266		1097		1066		907	
>12 hours/w		2.5 [1.5;4.0]		2.3 [1.3;4.0]		3.1 [1.7;5.5]		2.8 [1.5;5.5]		2.2 [0.9;5.3]		1.8 [0.7;4.8]
1–12 hours/w		2.1 [1.6;2.8]		1.9 [1.4;2.5]		2.6 [1.9;3.8]		2.2 [1.5;3.3]		1.8 [1.2;2.8]		1.7 [1.0;2.8]
0 hours/w (ref)		1		1		1		1		1		1
Equivalised childhood household income ^b^	2331				1257				1074			
High (> 13.400 EUR)		1.5 [1.1;2.1]				1.4 [0.9;2.1]				1.8 [1.1;2.9]		
Medium (10.980–13.400 EUR)		1.1 [0.8;1.5]				0.9 [0.6;1.3]				1.4 [0.9;2.2]		
Low (<10.980 EUR) (ref)		1				1				1		
Parents highest edu. level ^c^	2331				1259				1072			
>13 years		2.3 [1.6;3.3]				2.0 [1.2;3.1]				2.8 [1.7;4.8]		
10–13 years		2.4 [1.7;3.4]				2.0 [1.3;3.2]				3.0 [1.7;5.0]		
<10 years (ref)		1				1				1		
Subjective Social Status ^d^	2105				1144				961			
High		2.0[1.4;2.9]				1.7 [1.1;2.9]				2.3 [1.4;4.0]		
Medium		1.3 [0.9;1.9]				1.0 [0.6;1.7]				1.9 [1.0;3.5]		
Low (ref)		1				1				1		
Chronic disease	2352				1270				1082			
No		1.9 [1.4;2.5]				1.8 [1.2;2.7]				1.8 [1.1;3.0]		
Yes (ref)		1				1				1		
Depressive symptoms	2348				1271				1077			
Absent		1.7 [1.4;2.2]				1.6 [1.1;2.2]				1.9 [1.3;2.8]		
Present (ref)		1				1				1		
Self-rated Health	2355				1271				1084			
Good		1.7 [1.4;2.2]				1.8 [1.3;2.5]				2.4 [1.6;3.5]		
Poor (ref)		1				1				1		

^) Model: adjusted for equivalated childhood income, parents’ highest educational level, subj. social status, chronic diseases, depressive symptoms and self-rated health.

^a)^ Engagement in 2 different types of LTA daily or weekly per week

^b)^ Childhood equivalised household income categorized at the 33^rd^ and the 67^th^ percentile of the source population

^c)^ Highest completed educational level of the biological parents when each participant was 7–10 years old.

^d)^ Measured in 2004.

In both study populations, correlation analyses were carried out for all covariates using Pearson’s rank correlation coefficients. The tests showed no sign of multicollinearity (results not shown). The strongest correlation was between depressive symptoms and self-rated health with r = 0.26 at both time points. PTW and LTA were only slightly correlated (r = 0.094 (2004) and r = 0.064 (2007)). Furthermore, there was no interaction between PTW and LTA in any of the study populations.

Logistic regression models were used to estimate the associations between engagement in society in mid or late adolescence and connection to education and employment at the age of 25–29 years. The adjusted analyses were subsequently stratified by gender to examine a possible gender difference. Results were presented with crude and adjusted odds ratios (OR) and 95% confidence intervals (95% CI) (Tables 2 and 3, Adj. models). The adjusted models were stratified by parental educational level and subjective social status to investigate the strength of the associations across different socioeconomic groups and measures of socioeconomic position (Tables 4 and 5)

**Table 4:** Connection to education and employment in early adulthood according to engagement in society in mid adolescence, stratified by childhood social status.

Stratification by parental educational level^				Stratification by subjective social status*			
	Educational level <10 years	Educational level 10–13 years	Educational level >13 years		Low subjective social status	Medium subjective social status	High subjective social status
	n = 371	n = 1400	n = 1168		n = 409	n = 726	n = 1836
LTA	1.4[0.7;2.9]	2.0[1.3;3.2]	1.3[0.8;2.1]	LTA	1.9[0.9;4.0]	3.0[1.5;6.0]	1.2[0.8;1.7]
PTW	1.3[0.7;2.5]	1.6[1.1;2.3]	1.9[1.3;2.9]	PTW	2.1[1.1;3.7]	1.4[0.9;2.4]	1.6[1.1;2.3]
>12 hours/w	7.5[0.9;61.8]	1.7[0.8;3.6]	1.5[0.6;3,5]	>12 hours/w	2.3[0.6;8.6]	1.5[0.5;4.2]	1.9[0.9;4.0]
1–12 hours/w	1.2[0.6;2.3]	1.6[1.0;2.4]	2.1[1.3;3.2]	1–12 hours/w	2.0[1.1;3.6]	1.5[0.9;2.5]	1.6[1.1;2.3]

^ Model: adjusted for childhood income, subjective social status, chronic diseases in 2004, depressive symptoms in 2004 and self-rated health in 2004.

* Model: adjusted for childhood income, parents’ highest educational level, chronic diseases in 2004, depressive symptoms in 2004 and self-rated health in 2004.

**Table 5:** Connection to education and employment in early adulthood according to engagement in society in late adolescence, stratified by childhood social status.

Stratification by parental educational level¤				Stratification by subjective social status °			
	Educational level <10 years	Educational level 10–13 years	Educational level >13 years		Low subjective social status	Medium subjective social status	High subjective social status
	n = 269	n = 1107	n = 955		n = 284	n = 522	n = 1299
LTA	2.2 [0.8;4.3]	1.1 [0.5;2.1]	1.7 [0.8;3.6]	LTA	5.1 [0.6; 39.7]	0.9 [0.4;1.9]	1.6 [0.8;3.1]
PTW	2.3 [1.0;5.0]	1.7 [1.1;2.6]	2.1[1.3;3.2]	PTW	1.3 [0.7;2.6]	2.9 [1.7;5.1]	1.7 [1.2;2.6]
>12 hours/w	2.4 [0.5;12.3]	1.7 [0.8;3.7]	2.9 [1.2;7.1]	>12 hours/w	3.3 [0.7;16.1]	2.5 [0.96;6.4]	2.1 [0.99;4.3]
1–12 hours/w	2.2 [0.98;5.1]	1.7 [1.1;2.7]	1.9 [1.2;3.1]	1–12 hours/w	1.1 [0.5;2.2]	3.0 [1.7;5.5]	1.7 [1.1;2.5]

^¤^ Model: adjusted for equivalised childhood income, subjective social status, chronic diseases in 2007, depressive symptoms in 2007 and self-rated health in 2007.

° Model: adjusted for equivalised childhood income, parents’ highest educational level, chronic diseases in 2007, depressive symptoms in 2007 and self-rated health in 2007.

Data analyses were performed using STATA statistical package (version 15.0; Stata, College Station, TX, USA).

### Ethics

Use of the data was carried out under the same conditions and with the same purpose as originally collected. The study was reported to and approved by the Danish Data Protection Agency (journal no. 2012-58-006), according to Danish law for studies using questionnaire and register data. The approval was first given in 2003, prior to the initiation of the data collection, and has subsequently been renewed several times. Written informed consent was not required in questionnaire or register-based projects (The Act on Processing of Personal Data—Act No. 429 of 31 May 2000). This, whether participants were legally minors or adults.

Parents had the option to waive their children’s participation in the survey in 2004 and thus refrain from future participation in the survey. All medical information about minors were disclosed by their parents.

## Results

Table 1 shows that the 2004 study population consisted of an equal number of women and men, whereas the 2007 study population consisted of more women (54%). In both study populations, small differences in low connection to education and employment in early adulthood were seen between genders. More women had low connection (13–15%) compared to men (11%). Compared to mid adolescence where women and men were almost equally engaged in PTW, in late adolescence, more women (66%) than men (45%) had PTW and worked more hours. Both genders were equally engaged in leisure activities throughout adolescence but with a notable decrease in high engagement from 34% in mid adolescence to 13% in late adolescence. More women reported depressive symptoms, low self-rated health, and low subjective social status in both study populations.

Tables 2 and 3 show that high levels of PTW and LTA in mid and late adolescence were associated with connection to education and work in early adulthood. For all socioeconomic position measures, the strength of the associations increased with higher socioeconomic position (Tables 2 and 3). The crude associations between socioeconomic position variables and a positive connection to education and employment were more profound for men than women (crude analyses, Tables 2 and 3).

Across the analyses of associations between PTW in mid and late adolescence, and positive connection to education and work an almost twofold increase at both time points was seen (Tables 2 and 3). LTA in mid adolescence was associated with a positive connection to education and work (OR:1.6 [1.2;2.1]) (Table 2).

Among women, PTW in both mid and late adolescence was associated with an increase in positive connection to education and work (adjusted OR between 1.7 and 2.3). When applying PTW as a categorical variable based on work hours in three levels, similar associations were seen in both periods. However, in mid adolescence, the strongest association was seen for PTW 1–12 hours/week (OR: 1.7 [1.2; 2.4]), whereas in late adolescence, the strongest association was seen for PTW >12 hours/week (OR: 2.8 [1.5;5.5]).

Among men, PTW in mid or late adolescence were both associated with a positive connection to later education and work. When applying PTW as a categorical variable the strongest association was seen for PTW >12 hours/week in mid adolescence (OR: 2.2 [1.0;4.9]).

When stratifying the analyses on childhood socioeconomic position, we found varying but positive associations between engagement in society and positive connection to education and work for all strata in both mid adolescence (Table 4) and late adolescence (Table 5). Participants who in mid adolescence considered themselves to have a low social status appeared to benefit the most from engagement in society. In late adolescence, the estimates were strongest for participants from homes with lower educational level. However, the number of participants with high engagement in LTA was small (n = 297) resulting in small strata and thus imprecise estimates.

## Discussion

In this prospective study, we found that engagement in society in mid and late adolescence increased the connection to education and work in early adulthood. The strongest and most consistent associations were seen between PTW and connection to future education and work. The timing of engagement in society seems to have different impacts for women and men. LTA and PTW in mid adolescence were associated with a positive connection for both genders. For women, LTA and PTW in late adolescence were associated with a positive connection in early adulthood. For men, PTW were associated with a positive connection in early adulthood.

The findings from this study are similar to a study of young non-western Danish adults, which reported that PTW at the age of 13–16 was associated with a 40% increase in employment at the age of 25 [58]. Our results are likewise in line with the findings from Lesner et al. [19] who found that students who had 20–30 hours of PTW per month during the school year achieved better exam grades and enrolled faster into secondary education [19]. Outcomes in their study were measured as more immediate effects of PTW compared to our study, and they did not investigate if it eventually prevented disconnection from education or employment in adulthood.

In contrast to Lesner et al. [19], who showed positive outcomes of a moderate number of work hours, this study observed the strongest associations for participants working more than 12 hours/week. A cut point of 15–20 hours of work per week during the school year has been proposed in several studies [21, 24, 26] as the threshold where the negative consequences of PTW outweigh the positive. Mortimer [21] has identified that those working steadily a few hours per week most of a school year have the most beneficial outcomes such as improved management skills. Due to the a priori decision about the categorization of work hours, it is not possible to examine if participants working many hours have lower connection to education and employment later in life.

The strong association between working >12 hours/week and a positive connection to education and employment in early adulthood is interesting in the light of recent Danish educational policies. Since 2014, the Danish primary and lower secondary school has gone through a large reform. Pupils in mid adolescence have 35 lessons per week and now have the longest school days in Europe [59]. This study uses data from 2004 and 2007, prior to the reform in 2014, so the positive results found here come from a period where adolescents had more spare time. The longer school days make it difficult for adolescents to have enough time for both obligatory homework, LTA and PTW, particularly because the Working Environment authorities only allow adolescents to work 2 hours per school day and 7–8 hours on non-school days [46]. The results of this study are relevant to policymakers when discussing future educational policies.

In both study populations, more women were disconnected in early adulthood. This is in contrast to Benjaminsen and colleges [9] who identified more disconnected men. This discrepancy is interesting as women in the present study worked more in late adolescence and the association between PTW and positive connection was strongest for women. The different results of the two studies can partly be explained by the fact that in Denmark, more men in late adolescence work in apprenticeships or vocational education [60], why fewer men have PTW during upper secondary education compared to women.

Another possible explanation for the difference can be related to the period where connection is measured since many young people establish families between the ages of 25–29. Although our data takes maternity leave into account, it is not possible to identify pregnancy-related sickness leave in the DREAM database [44]. Absenteeism due to pregnancy complications are registered as ordinary sickness and it is possible that some women were not truly disconnected, but rather on sick leave because of complications due to pregnancy. As a sensitivity analysis, register information about all childbirths from the female participants was collected between age 25 and 28. Unfortunately, data was unavailable up to the age of 29 years at the time of analysis. All recorded sickness benefits six months prior to childbirth were subtracted from the total number of weeks of received social benefits. A total of 10 women had been categorized with low connection to education and employment instead of high. These women were recoded, and all analyses repeated, which revealed only slight changes to the estimates (results not shown).

The univariate analyses indicate a social gradient, with participants from higher socioeconomic position having a higher connection to education and employment. Studies within the same cohort have previously identified a social gradient in dropout from high school [61] and levels of perceived stress [62]. However, the multivariate analyses stratified by socioeconomic position measures show that the associations between both LTA and PTW and positive connection to education and work vary within strata depending on the type of engagement, timing, and measure of socioeconomic position. Some of the strata were small, making the associations ambiguous and caution is advised in relation to interpretation. The present findings indicate that engagement in society is associated with a positive connection across different socioeconomic groups. However, in line with previous research, we did not find a compelling gradient when exploring the association between engagement in society and connection to education and work [63]. Thus, if LTA or PTW are to be used as promotional strategies to improve connection to education and work, considerations ought to include the definition of the target group and the timing of implementation.

### Strengths and limitations

A strength of this study is the use of a complete regional cohort with a relatively high initial participation rate in combination with high-quality register data. The DREAM register data was complete for all participants. The prospective study design allowed for an evaluation of temporal associations between LTA or PTW and later educational attainment and employment. With up to 14 years of follow-up in the study, the design made it possible to ensure that almost all of the participants had participated in society through education and employment. Positive connection to education and work was measured over four years to account for sporadic periods of disconnection. The use of both questionnaire and register-based data in the study minimises the risk of common method variance [64].

The study population was a young population, who often do not have as stable a labour market connection as older age-groups. Low connection to education and employment was defined, as more than 52 weeks without connection during four consecutive years. It was difficult to decide exactly for how long a person needed to be without connection to be genuinely disconnected from society. To examine an alternative cut point a sensitivity analysis with a cut point of 26 weeks without connection was performed. The sensitivity analysis showed that the relative estimates either decreased or remained the same size (results not shown).

The exposures in the study were constructed from questionnaires. The decision to participate was taken without any knowledge of subsequent outcomes in the study and we assume selection on participation was non-differential, which most likely will not bias the estimates. Based on the available register information, analyses of non-participation in the two study populations compared to the source population show that in both waves, more women have responded, and a higher proportion of the responders come from families with high income and educational levels. However, a previous study on the same cohort has shown that neither non-participation nor loss to follow-up influences the size or directions of the measured exposure-outcome associations significantly [41].

Self-reported data is prone to information bias if participants intentionally or accidentally respond incorrectly resulting in misclassification of variables. Questions on PTW and LTA are not regarded as personal questions such as e.g. health information [65]. We do not expect any misclassification to be associated with the outcome, thus any bias would most likely lead to an attenuation of the relative estimates. Most adolescents in Denmark are employed on an hourly basis, which increases the chance that the reported work hours are true. Yet, the number of hours adolescents work is likely to fluctuate during the school year, which the questionnaires were unable to capture, possibly resulting in non-differential information bias.

Overall, the limitations identified are not considered to cause serious bias in relation to the associations found in the study. However, caution about causal interpretation is important since other factors may have confounded the associations, such as ambition, self-confidence, indomitability, or “believing”, which have been found to impact both societal engagement and positive connection to education and work [66, 67].

Previous research by Glasscock et al. [62] has shown that the socio-demographical characteristics of the participants of the Vestliv Cohort are comparable to young people in the rest of Denmark. Many participants in Vestliv have moved and are now living in different parts of Denmark reflecting various possibilities for education and employment. Therefore, the findings from this study are generalisable to young people in Denmark and other countries with similar environmental and social conditions, taking into consideration that Danish adolescents today have longer school days making the balance between school and leisure time more difficult for pupils now than for the participants of this study.

## Conclusion

This study shows a notable association between adolescent engagement in society and a positive connection to education and work in early adulthood, with stronger effects of PTW compared to LTA. Furthermore, the study identifies a small gender difference in both the association between societal engagement on positive connection to education and work and in the timing of adolescent engagement. The associations between engagement in society and positive connection vary across socioeconomic groups and depend on the chosen indicator for socioeconomic position. Municipalities, social workers, and public health workers need to be more attentive to the positive effects of adolescent engagement in society to reduce the risk of disconnection in the transition into adulthood.

From a perspective of public health, it is essential to understand the factors associated with a positive connection to education and work to develop strategies, which are effective in terms of prevention and match the needs of different groups. The findings in this study indicate that establishment of easier access to leisure time activities and an increase in the number of part-time job positions for adolescents both appear to be relevant and feasible preventive strategies.
